# A Freeze-Dried Cranberry Powder Consistently Enhances SCFA Production and Lowers Abundance of Opportunistic Pathogens In Vitro

**DOI:** 10.3390/biotech11020014

**Published:** 2022-05-06

**Authors:** Christina Khoo, Cindy Duysburgh, Massimo Marzorati, Pieter Van den Abbeele, Derek Zhang

**Affiliations:** 1 Ocean Spray Cranberries, Inc., Bridge Street 152, Middleborough, MA 02349, USA; 2 ProDigest BV, Technologiepark-Zwijnaarde 73, 9052 Ghent, Belgium; cindy.duysburgh@prodigest.eu (C.D.); massimo.marzorati@prodigest.eu (M.M.); 3 Center of Microbial Ecology and Technology (CMET), Ghent University, 9000 Ghent, Belgium; 4 Cryptobiotix SA, Technologiepark-Zwijnaarde 82, 9052 Ghent, Belgium; pieter.vandenabbeele@cryptobiotix.eu; 5 IQVIA, Emperor Boulevard 4820, Durham, NC 27703, USA; derek.zhang@iqvia.com

**Keywords:** SCFA, cranberry, prebiotic, in vitro, pathogen, *Akkermansia muciniphila*

## Abstract

The American cranberry, *Vaccinium macrocarpon*, contains fibers and (poly)phenols that could exert health-promoting effects through modulation of gut microbiota. This study aimed to investigate how a freeze-dried whole cranberry powder (FCP) modulated metabolite production and microbial composition using both a 48-h incubation strategy and a long-term human gut simulator study with the M-SHIME (Mucosal Simulator of the Human Intestinal Microbial Ecosystem). FCP was repeatedly administered over three weeks. The studies included five and three study subjects, respectively. In both models, FCP significantly increased levels of health-related short-chain fatty acids (SCFA: acetate, propionate and butyrate), while decreased levels of branched-chain fatty acids (markers of proteolytic fermentation). Interestingly, FCP consistently increased luminal *Bacteroidetes* abundances in the proximal colon of the M-SHIME (+17.5 ± 9.3%) at the expense of *Proteobacteria* (−10.2 ± 1.5%). At family level, this was due to the stimulation of *Bacteroidaceae* and *Prevotellaceae* and a decrease of *Pseudomonodaceae* and *Enterobacteriaceae*. Despite of interpersonal differences, FCP also increased the abundance of families of known butyrate producers. Overall, FCP displayed an interesting prebiotic potential in vitro given its selective utilization by host microorganisms and potential health-related effects on inhibition of pathogens and selective stimulation of beneficial metabolites.

## 1. Introduction

Diet is an established modulator of the gut microbiota [1], with plant-based foods being increasingly linked to health-promoting effects. Plant-based diets have been shown to stimulate levels of beneficial short-chain fatty acids (SCFA) and beneficial microorganisms (e.g., *Bifidobacterium* and *Lactobacillus* species), while lowering the abundances of microorganisms that can trigger diseases [2,3]. Moreover, the number of bioactive plant species in the diet is an important predictor of microbial diversity [4], loss of which seems to be a general feature in western diets [5] and in cases of diseases [6].

The American cranberry, *Vaccinium macrocarpon*, contains different nutrients with an interesting potential for shifting the gut microbiota towards a health-promoting profile. First, cranberries contain indigestible and fermentable dietary fiber, a key nutrient class that drives the energy and carbon utilization in the colon [7]. Secondly, the American cranberry is a rich source of (poly)phenols with anticarcinogenic, anti-inflammatory and antioxidant properties [8], and is therefore proposed to limit the development and severity of certain chronic diseases [9]. Dietary polyphenols can be strong modulators of the human gut microbiota, as over 95% of the intake passes to the colon unabsorbed [10]. For example, epidemiologic studies repeatedly identify polyphenol-rich products such as red wine, coffee and tea as stimulators of gut microbial diversity [11,12]. Few studies have investigated the impact of cranberries on gut microbiota. In a mice study, Anhê et al. (2015) [13] showed that a polyphenol-rich cranberry extract specifically stimulated *Akkermansia* spp., and thereby protected against diet-induced obesity, insulin resistance and intestinal inflammation. In our previous in vitro work, a cranberry concentrate was shown to suppress adherent-invasive *Escherichia coli* (AIEC) as it decreased its adhesion and invasion in colon mucosal and epithelial models [14]. In a placebo-controlled study in women suffering from recurrent urinary tract infections, long-term daily consumption of cranberry juice decreased the abundance of *Flavonifractor*, a species that has been associated with negative health effects [15]. The potential cranberries to exert beneficial effects on human health via the modulation of human gut microbiota suggests that more research is to be carried out in order to better understand the underlying changes.

During each of the aforementioned studies focusing on the impact of cranberries on gut microbiota, different fractions or extracts of cranberries were used. Previously, it was demonstrated that whole cranberry powder had a stronger impact on simulated colon microbiota than various cranberry fractions [16]. Phenol as well as non-phenol compounds of cranberries, such as salicylate, reduced *Enterobacteriaceae* and promoted *Bacteroidaceae*, but cranberry powder caused the greatest *Enterobacteriaceae* reduction. This suggests that the different nutrients of cranberries may act synergistically.

Therefore, we aimed to investigate the effects of a freeze-dried whole cranberry powder (FCP) on health-promoting parameters of microbes in vitro. Upon showing promising effects on SCFA production and *Bifidobacteriaceae* levels in an exploratory short-term fecal incubation experiment (with five study subjects), we went on to investigate repeated administration of FCP in a M-SHIME model (as tested for three study subjects). As the M-SHIME also comprises a mucosal compartment, it simulates adhesion of pathogens [17] and the mucin-specific microbiota [18]. Our study is the first to investigate the impact of FCP on an in vitro-simulated mucosal microbiota.

## 2. Materials and Methods

### 2.1. Chemicals

All chemicals were obtained from Sigma (Bornem, Belgium) unless otherwise specified. FCP was supplied by The Cranberry Institute (Varver, MA, USA) and its composition was characterized (Table 1).

### 2.2. Incubation Strategy—Test 1

**Figure 1:**
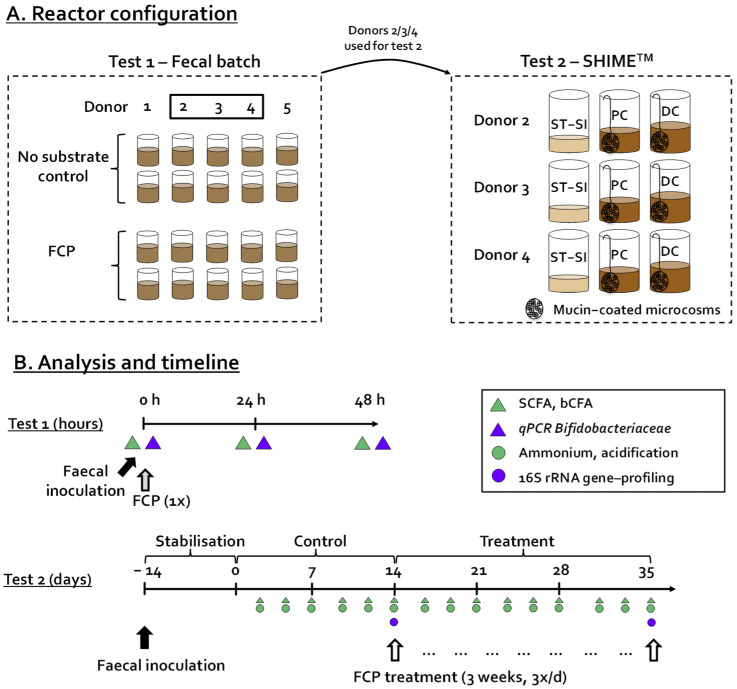
(**A**) Reactor configuration and (**B**) timeline of the in vitro experiments (test 1 and test 2) during which the impact of a freeze-dried cranberry powder (FCP) was assessed on the activity and composition of the simulated human colonic microbiota. SHIME, Simulator of the Human Intestinal Microbial Ecosystem; ST-SI, stomach–small intestine; PC, proximal colon; DC, distal colon; bCFA, branched-chain fatty acids; SCFA, short-chain fatty acids.

Faecal incubation of 48 h was performed as published previously [19], allowing the assessment of the impact of FCP on the simulated human colonic microbiota as compared to a no-substrate control (blank) of Belgian human adult donors 1 (m, 25 yr), 2 (m, 27 yr), 3 (f, 24 yr), 4 (m, 32 yr) and 5 (f, 34 yr). All donors were healthy with a normal Body Mass Index (BMI) and did not use antibiotics during the last six months prior to this study. As all incubations were performed in technical duplicate, this resulted in a set-up with 20 independent reactors (Figure 1A). Briefly, the incubation strategy involved following steps. First, 63 mL of nutritional medium buffered at pH = 6.5 (K_2_HPO_4_, 3.5 g/L; KH_2_PO_4_, 10.9 g/L; NaHCO_3_, 2 g/L; yeast extract, 2 g/L; peptone, 2 g/L; starch, 2 g/L; mucin, 1 g/L; cysteine, 0.5 g/L; and Tween 80, 2 mL/L) was added to 120 mL penicillin bottles, containing the correct amount of different test products to reach a final concentration of 5 g/L test product (corresponding to 140 mg/L polyphenols). After adding the nutritional medium, the bottles were closed with butyl rubber stoppers and flushed with nitrogen to render them anaerobic. At that point, 7 mL of a fecal slurry was administered. The slurry was prepared by mixing 7.5 g fecal sample with 100 mL anaerobic phosphate buffer (K_2_HPO_4_, 8.8 g/L; KH_2_PO_4_, 6.8 g/L; sodium thioglycolate, 0.1 g/L; and sodium dithionite, 0.015 g/L), homogenization (10 min, BagMixer 400, Interscience, Louvain-La Neuve, Belgium) and finally removal of large particles via centrifugation (500× *g* for 2 min).

During the 48-h incubation period, simulating a single intake of FCP, the temperature was controlled at 37 °C, while the bottles were continuously agitated (90 rpm). Samples were collected for the analysis of microbial metabolic activity (SCFA and bCFA) and the presence of *Bifidobacteriacae* was quantified by qPCR at 0 h, 24 h, and 48 h (Figure 1B).

### 2.3. Incubation Strategy—Test 2

The SHIME^®^ technology (ProDigest, Ghent University, Ghent, Belgium) was used to conduct test 2. The reactor design was modified to allow parallel comparison of fermentation by three fecal samples, leading to a triple-M-SHIME setting, i.e., three parallel units, each consisting of three sequential reactors (Figure 1A). The first reactor was a combined stomach-small intestinal reactor (ST-SI) operated according to the fill-and-draw principle. Subsequently, the suspension was transferred to a simulated proximal colon (PC) (500 mL/pH between 5.7–5.9) and distal colon (DC) (800 mL/pH between 6.6–6.9). pH was adjusted by the addition of 0.5 M NaOH and 0.5 M HCl. Besides a liquid phase, each colonic reactor also contained 60 mucin-coated microcosms (AnoxKaldnes K1carrier, AnoxKaldnes AB, Lund, Sweden) prepared according to Van den Abbeele et al. [18]. Half of the mucin beads were renewed three times per week by opening the reactors as described recently [17]. All reactors were airtight, continuously agitated (300 rpm), and temperature controlled (37 °C).

In terms of timeline, test 2 consisted of a stabilization (day −14 to 0), control (day 0 to 14) and FCP treatment period (day 14 to 35) At the start of the experiment, all colonic reactors were filled with 95% SHIME nutritional medium (ProDigest, Belgium) and 5% of a fresh fecal slurry of donors 2, 3, and 4 (prepared by mixing 20 g fecal sample with 100 mL anaerobic buffer, followed by homogenization and centrifugation as described above for test 1). Anaerobiosis was maintained by flushing the headspace of all reactors with nitrogen gas (Parker Balston Nitrogen Generator UHPN2-1100, CompAir Geveke, Vilvoorde, Belgium). After overnight incubation, three feeding cycles were applied and each consisted of an administration of 140 mL of SHIME nutritional medium (simulated gastric phase) and 60 mL of bile/pancreatic fluid (simulated small intestinal phase). While the SHIME^®^ was operated according to its standard parameters during the stabilization and control period (day −14 to 0 and day 0 to 14), FCP was added at 7.14 g/L in the nutritional medium between days 14 to 35 (Figure 1B). Upon repeated administration (3 times per day) and upon dilution with bile/pancreatic juice during each feeding cycle (140/200 ratio), this would result in a theoretical maximal concentration of 5 g/L in the PC and DC region.

To avoid potential issues with stability of the in vitro microbiota over time, quality control was performed consisting of weekly verification of (i) the accuracy of pumped volumes of pumped nutritional medium and bile/pancreatic fluid (via a mock feeding cycle), and (ii) the accuracy of measured pH values by the SHIME instrument (by measuring collected samples of all reactors with an external pH probe (Senseline pH meter F410 (ProSense, Oosterhout, The Netherlands)).

### 2.4. Microbial Metabolic Activity

During test 1 and 2, short-chain fatty acids (SCFA; acetate, propionate and butyrate) and branched-chain fatty acids levels (bCFA: sum of isobutyrate, isovalerate and isocaproate) were measured via a GC-FID method described by De Weirdt et al. [20], upon performing a diethyl ether extraction (with use of 2-methyl hexanoic acid as internal standard). 

### 2.5. Microbial Composition Analysis

For liquid samples, 1 mL aliquots were centrifuged at 9000× *g* for 5 min. Upon discarding the supernatant, the DNA was extracted from the pellet as described by Boon et al. [21] with modifications implemented by Duysburgh et al. [22]. Similarly, 0.25 g samples of the simulated mucus layer were extracted as such.

During test 1, quantitative polymerase chain reaction (qPCR) was used to quantify *Bifidobacteriaceae*. Briefly, a StepOnePlus™ real-time PCR system (Applied Biosystems, Foster City, CA, USA), was used according to a previously described protocol [23]. The 5′-3′ and 3′-5′ primers were TCGCGTCYGGTGTGAAAG and CCACATCCAGCYTCCAC, respectively. The protocol started with 10 min incubation at 95 °C and terminated with a melting curve from 60 °C to 95 °C. 40 cycles were performed with a denaturation phase of 15 s at 95 °C, an annealing phase of 30 s at 60 °C, and an elongation step of 30 s at 72 °C in each cycle.

On specific samples collected during test 2, 16S-rRNA gene profiling was performed to obtain insights of changes at overall community level. Sequencing was performed by LGC Genomics GmbH (Berlin, Germany) as described recently [24]. The MiSeq protocol for read assembly and cleanup using the mothur software (v. 1.39.5) was adapted as described previously [25,26]. Briefly, reads were assembled into contigs, followed by alignment-based quality filtering, removing of chimeras and taxonomy assignment via a naïve Bayesian classifier [27] and RDP release 14 [28]. Finally, contigs were clustered into OTUs at 97% sequence similarity. Unclassified sequences at (super)Kingdom were removed together with sequences classifying as Eukaryota, Archaea, Chloroplasts and Mitochondria. Across samples, the total number of combined reads was on average 21,918 (minimum = 4579; maximum = 36,841). After filtering out OTUs with 5 or less than 5 reads across samples, the final number of combined reads was on average 13,260 (minimum 2390; maximum 35,638). Rarefaction curves were made with Past 4.03 [29] to confirm that the sequencing depth allowed to grasp the microbial diversity of samples of the inocula and in vitro samples.

### 2.6. Statistics

For exploratory data analysis, Principal Component Analysis (PCA) was performed for metabolic activity data (SCFA and bCFA) obtained during test 1 and 2 via GraphPad Prism version 9.2.0 (Graphpad Software, San Diego, CA, USA).

Further, for statistical analysis of treatment effects of FCP during test 1, repeated measures ANOVA with post-hoc Benjamini-Hochberg correction [30] was applied to assess differences between the no-substrate control and FCP across the 5 donors tested (FDR = 0.05). Treatment effect of FCP on SCFA production during test 2 was also assessed via a repeated measures ANOVA with post-hoc Benjamini-Hochberg correction between the samples collected during the final week of treatment of FCP (*n* = 3 per donor) as compared to the samples collected during the final control week (*n* = 3 per donor). Given that there were 3 donors, this resulted in a total of sample size of 9 (FDR = 0.05). Statistical differences were indicated by */**/*** (0.01 < *p* < 0.05/0.001 < *p* < 0.01/*p* < 0.001).

Finally, treatment effect of FCP on microbial diversity (reciprocal Simpson diversity index) and microbial composition at family level (test 2) was assessed via a repeated measures ANOVA with post-hoc Benjamini-Hochberg correction, comparing samples collected during the final week of treatment of FCP (*n* = 1 per donor) as compared to the samples collected during the final control week (*n* = 1 per donor) (FDR = 0.05). 

## 3. Results

### 3.1. A Single Dose of FCP Consistently Boosted SCFA and Lowered bCFA Levels during Fecal Batch Incubations of Five Human Adults (Test 1) 

**Figure 2:**
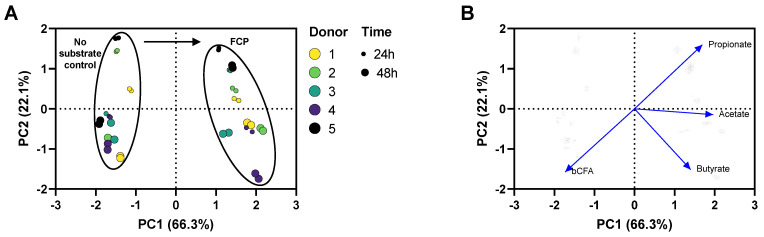
(**A**) PCA based on microbial metabolic activity on different time points (24 h and 48 h) during the incubation of human faecal microbiota of five different human donors in presence of FCP *versus* a no substrate control. All conditions were tested in technical duplicate. (**B**) Loadings of the parameters on which the PCA was based: acetate, propionate, butyrate, and bCFA. PCA, principal component analyis.

**Figure 3:**
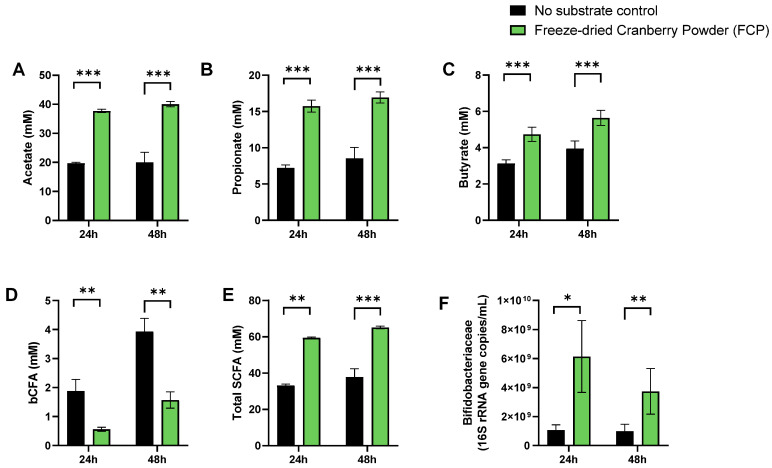
Effect of FCP on levels of acetate (**A**), propionate (**B**), butyrate (**C**), bCFA (**D**), total SCFA (**E**) and *Bifidobacteriaceae* (**F**), upon incubation with a human faecal microbiota derived of five different human donors. All conditions were tested in technical duplicate. The data are presented as the average value (±SEM) across all donors and replicates (*n* = 10). Statistical differences due to treatment were indicated by */**/*** (0.01 < *p* < 0.05/0.001 < *p* < 0.01/*p* < 0.001).

To gain insight of overall changes in microbial activity upon FCP treatment, a principal component analysis (PCA) was performed (Figure 2). Technical replicates clustered closely together demonstrating the high reproducibility of the assay. Further, the PCA analysis accounted for 88.4% of the observed variation of the dataset. Most of the variance was explained along PC1 (X-axis) linked with the differential clustering of the ‘no-substrate control’ versus FCP-treated incubations. FCP markedly increased the levels of acetate, propionate and butyrate (and thus total SCFA) while decreasing bCFA levels (Figure 2A–E). Further, 22% of the overall variation was explained along PC2 (Y-axis), mostly due to the additional butyrate and bCFA production between 24 h and 48 h. Finally, within clusters of treatment/time, minor interpersonal differences were observed. Overall, the impact of FCP on the simulated human gut microbiota was highly consistent across the five donors tested (Figure S1A–E). Finally, *Bifidobacteriaceae* levels significantly increased when averaged across the five different donors suggesting their involvement in FCP fermentation (Figure 3F).

### 3.2. Repeated Adminsitration of FCP Consistently Boosted SCFA and Lowered bCFA Levels in the Simulated Proximal and Distal Colon of Three Human Adults Tested in the Triple-M-SHIME (Test 2)

Given the consistent effects of a single dose of FCP on the simulated human gut microbiota during test 1, any of the five donors tested could have been used for test 2 during which the effect of repeated administration of FCP was investigated. To investigate potential interpersonal differences, it was opted to test FCP fermentation for more than one donor during test 2 (donors 2/3/4).

**Figure 4:**
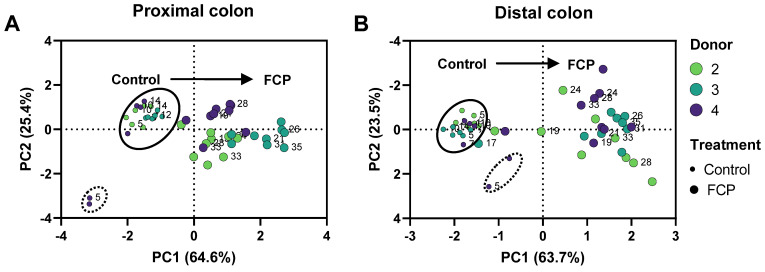
Principal component analysis (PCA) based on microbial metabolic activity in the proximal (**A**) and distal (**B**) colon during the control and treatment with FCP, as tested for three human adult donors 2, 3, and 4 in the triple-M-SHIME. The PCA is based on the individual values for each donor and for each time point (three samples per week during five experimental weeks: two control weeks (3/5/7/10/12/14) and three treatment weeks (17/19/21/24/26/28/31/33/35)). The following data were included: acetate, propionate, butyrate, and bCFA. Arrows indicate the evolution of samples along the duration of the experiment.

**Figure 5:**
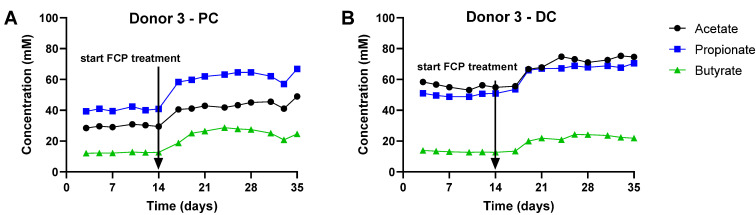
Effect of fermentation of FCP on SCFA levels (mM) in the simulated proximal colon (**A**) and distal colon (**B**) during both the control (Days 0–14) and treatment period (Days 14–35) of the triple-M-SHIME for donor 3. Results are representative of those obtained for Donors 2 and 4 (Supplementary Figure S1). Arrows indicate the start of the FCP treatment.

To gain insight into overall changes in microbial activity upon FCP treatment, the data were presented in two ways: (i) PCAs (Figure 4) and (ii) longitudinal SCFA levels (Figure 5 for donor 3 and Figure S2 for donors 2/4). PCAs explained a great percentage of the overall variation of the dataset, i.e., 90.0% and 87.2% for the PC and DC, respectively. Samples collected during the control period contained similar levels of SCFA, as visualized by stable lines in the longitudinal SCFA profiles (Figure 5) and by co-localization of samples of the control period in the PCAs (indicated by full line) (Figure 4). The only exception were samples collected on the first time points of the control period for donor 4 (days 3/5). Nevertheless, also for this SHIME unit, the simulated microbiota was fully stable at the end of the first control week onwards. 

**Figure 6:**
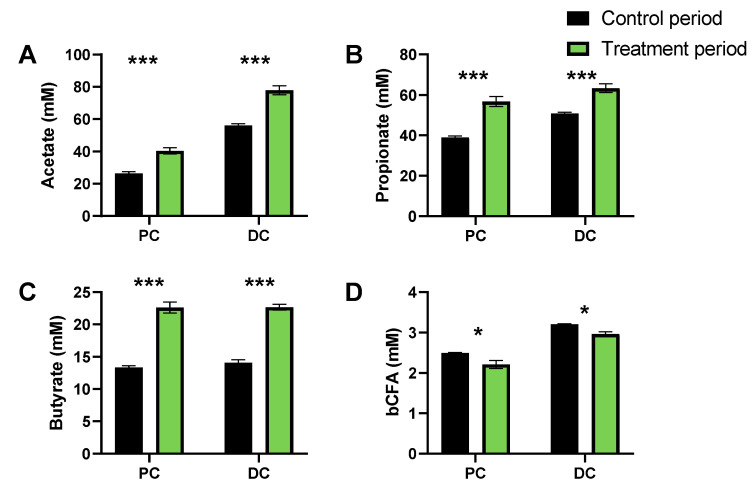
Effect of fermentation of FCP on microbial activity in the simulated proximal and distal colon of the triple-M-SHIME during both the final week of the control (Days 9–14) and treatment period (Days 30–35). The data is presented as the average value (±SEM) of acetate (**A**), propionate (**B**), butyrate (**C**) and bCFA (**D**), across the three samples collected per week for each of the three human adult donors tested (*n* = 9). Statistically significant differences between samples collected during the final control and treatment week are indicated by */*** (0.01 < adjusted *p*-value < 0.05/*p* < 0.001).

Most of the variation was thus explained along PC1, which related to the marked differential clustering of control samples (left) as opposed to samples collected during the FCP treatment period (right). This was confirmed by the marked increase of SCFA levels at the start of FCP treatment onwards (Day 17), for each of the three donors tested. The treatment effect of FCP became more pronounced during the first week of treatment, after which it reached a steady state. When treatment effect was compared between control and treatment from the final week, FCP significantly increased acetate, propionate and butyrate levels, while decreasing bCFA levels in both simulated colon regions (Figure 6).

### 3.3. Repeated Adminsitration of FCP Consistently Altered Microbial Composition in the Simulated Proximal and Distal Colon with Some Interpersonal Differences across the Three Human Adults Tested in the Triple-M-SHIME (Test 2)

**Figure 7:**
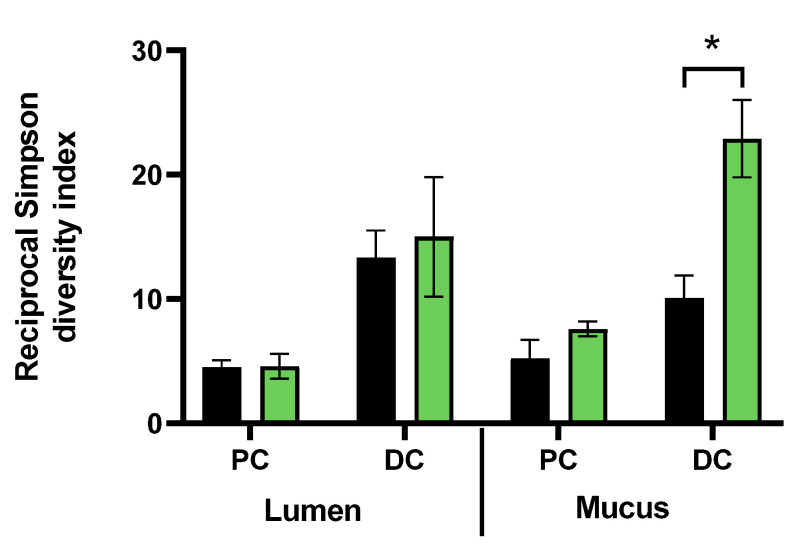
Effect of fermentation of FCP on average (±SD) microbial diversity (reciprocal Simpson diversity index) in the simulated proximal colon and distal colon of the triple-M-SHIME, at the end of the treatment period (TR: Day 35) and the end of the control period (C: Day 14) (*n* = 1 for 3 donors). Statistical differences due to treatment were indicated by * (0.01 < adjusted *p*-value < 0.05).

When microbial diversity in the lumen was evaluated, it remained unaffected. In contrast, FCP treatment significantly increased bacterial diversity from 10.1 ± 1.8 up to 22.9 ± 3.1 in the mucosal environment of the distal colon (adjusted *p*-value = 0.017) (Figure 7).

**Figure 8:**
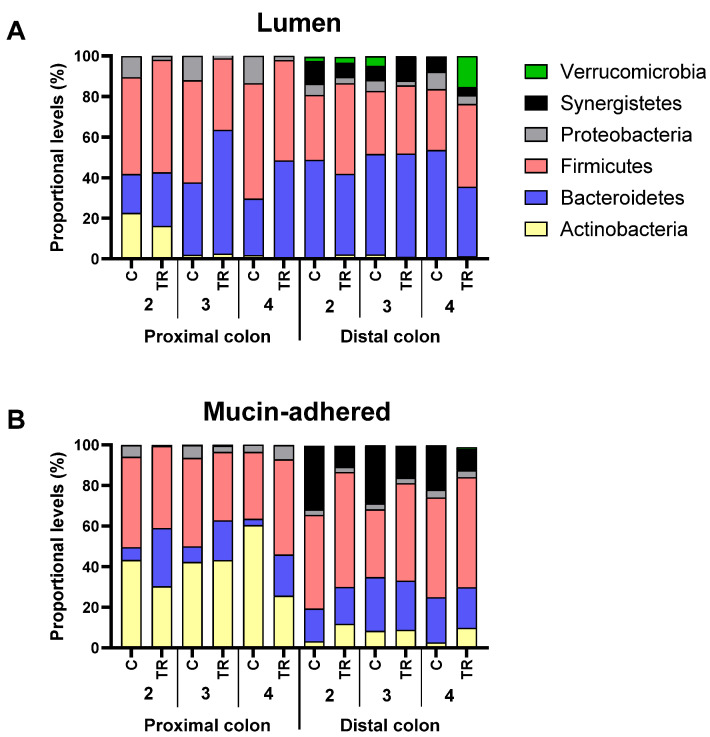
Effect of FCP on microbial composition (phylum level) in the lumen (**A**) and mucus (**B**) (as estimated via 16S rRNA gene profiling (%)) of the simulated proximal and distal colon of the triple-M-SHIME at the end of the control (C: Day 14) and treatment period (TR: Day 35) (*n* = 1).

When data was presented at phylum level (Figure 8), FCP treatment resulted in a consistent increase of *Bacteroidetes* levels in the proximal colon (+17.5 ± 9.3%) at the expense of Proteobacteria (−10.2 ± 1.5%). When focusing on intrinsic differences between the simulated lumen and mucus, it was noted that the simulated mucus was enriched with Actinobacteria and *Synergistetes/Firmicutes* in the proximal and distal colon, respectively.

Table 2 and Table S1 focus on the changes induced by FCP at family level of the lumen and simulated mucus, respectively. Positive values in the tables indicate that the abundance of a bacteria family was stimulated by FCP, while negative values suggest the inhibition. The composition of the luminal and mucosal microbiota at baseline is provided as supplementary information (Table S2 and Table S3). First, the consistent increase of *Bacteroidetes* at family level in the proximal colon was due to the increase of *Bacteroidaceae* and *Prevotellaceae*, for donor 2 and donors 3/4, respectively. The increase was mostly at the expense of *Veillonellaceae* and various Proteobacteria families (*Enterobacteriaceae*, *Pseudomonadaceae* and *Xanthomonadaceae*). Furthermore, FCP also increased the abundance of *Acidaminococcaceae*. In the distal colon, FCP increased the levels of *Lachnospiraceae* and *Ruminococcaceae*. For donor 4, a remarkable increase of *Verrucomicrobiaceae* was noted in the lumen of the distal colon upon FCP treatment.

In the mucosal environment, treatment effect was subject to interpersonal differences. Nevertheless, the main findings of the luminal microbiota were confirmed, i.e., increase of *Bacteroidaceae* (donors 2/3) and *Prevotellaceae* (donors 3/4) in the proximal colon, together with stimulation of *Acidaminococcaceae* in both simulated colon regions. Other consistent changes were the decrease of *Synergistaceae* and *Desulfovibrionaceae* in the distal colon upon FCP treatment. Donor-specific treatment effect of FCP included an increase of *Lachnospiraceae* (donors 2/3), *Erysipelotrichaceae* (donor 4) and *Bifidobacteriaceae* (donors 2/4).

## 4. Discussion

A freeze-dried whole cranberry powder (FCP) consistently modulated colon microbial communities towards a more health-promoting profile in our in vitro experiments. FCP significantly promoted SCFA production in both phases of the current study. A single dose of FCP (Figure 3) as well as its repeated administration (Figure 5) significantly increased acetate, propionate and butyrate levels. The concomitant decrease in branched SCFA production further indicated that FCP steered the colon microbial activity away from potentially toxic, proteolytic activity [31]. This is our second report of a directional stimulatory effect of cranberries towards acetate and propionate in vitro [14]. Acetate and propionate are widely recognized for their health benefits, and exert metabolic and anti-inflammatory effects even far beyond the gut, in peripheral tissues such as the liver, brain, adipose tissue, pancreas and muscles [32]. The marked stimulation of *Bacteroidetes* members (*Bacteroidaceae* and *Prevotellaceae*), able to produce acetate and propionate (via the succinate pathway [33]), explained the potent increases of acetate and propionate. The consistent increase of *Acidaminococcaceae*, a family containing species such as *Phascolarctobacterium faecium*, an abundant gut colonizer [34] that is able to convert succinate into propionate [35], further supports the potent effect of FCP on the increase of propionate. For butyrate, the primary site of sequestration is the gut epithelium, where it is the preferred fuel for colonocytes [36,37]. A rather limited number of organisms, belonging to *Lachnospiraceae* and *Ruminococcaceae* (among others *Faecalibacterium prausnitzii*), are responsible for the major fraction of butyrate production [38], and they offer benefit to the mucosal environment, i.e., M-SHIME, for their successful colonization in vitro [18]. Previously, we found that cranberries were able to stabilize *Lachnospiraceae* and *Ruminococcaceae* populations in the absence of a simulated mucosal environment [16]. In the present study, repeated dosing of FCP in the M-SHIME model had a pronounced effect on butyrate production despite interpersonal effects on butyrate-producing families. Overall, based on the aforementioned findings, we hypothesize that cranberries primarily and directionally act on acetate and propionate-producing species belonging to the *Bacteroidetes* phylum, and stimulate butyrate-producing species indirectly for example by acting on cross-feeding or inhibition of competing species.

FCP dosing to the M-SHIME model confirmed a potent inhibitory effect of cranberry-derived products towards microorganisms that are often associated with diseases with the potential to adhere to or invade host cells [39,40,41,42]. In the mucosal compartment, FCP reduced levels of *Desulfovibrionaceae* and *Synergistetes*, while stimulating *Acidaminococcaceae*. Both *Synergistetes* and *Acidaminococcaceae* are amino acid-degrading bacteria [43], indicating that FCP dosing resulted in a competitive advantage for members of the *Acidaminococcaceae*. *Acidaminococcaceae* contribute to propionate production in the gut [33,35]. Changes in the abundances of *Acidaminococcaceae* have been associated with depression, but both positively and negatively, so that their contribution to human health remains unclear [44]. In contrast, *Synergistetes* are considered to be opportunistic pathogens [43]. They have been recovered from pathophysiological conditions, such as periodontitis [45]. Intestinal *Desulfovibrionaceae* are hydrogen sulfide gas producing species, a potentially toxic feature which is associated with the pathophysiology of inflammatory bowel diseases and colorectal cancer [46]. Further, in the luminal compartment, FCP also decreased members of the *Proteobacteria* family, with reductions in *Pseudomonodaceae* and *Xanthomonodaceae* in the distal colon and a remarkable reduction in *Enterobacteriaceae* in the proximal colon. *Pseudomonodaceae* is a family of motile bacteria, comprising *Pseudomonas aeruginosa*, a multidrug-resistant species that causes intestinal infection in hospitalized an immune-compromised patients [47]. *Xanthomonodaceae* harbor mostly bacterial phytopathogens but also human multidrug resistant *Stenotrophomonas* species, which cause nosocomial infections in immunodeficient patients [48,49]. Finally, the *Enterobacteriaceae* family is a large family harboring many intestinal (opportunistic) pathogens, such as *Salmonella*, *Shigella* and *Escherichia coli*. This is our third report of a directional effect of cranberries towards lowering the abundances of *Enterobacteriaceae* in various in vitro models of the human colonic microbiota [14,16].

Interestingly, FCP dosing strongly increased biodiversity in the distal mucosal compartment. Gut microbiotas are less biodiverse among western population [5], and appear to have a lower metabolic potential when compared to those of individuals living in agrarian societies [50]. More specifically, indigestible carbohydrates are converted to lower levels of SCFA [50]. Loss of biodiversity is a common feature in cases of diseases [6]. Previously, long-term dosing of cranberry fractions improved alpha-diversity and microbial richness in mice fed a western-style diet [51]. A higher biodiversity upon FCP dosing in the M-SHIME has emerged as an important outcome of this study, motivating further study towards the therapeutic potential for an improved western gut microbiome.

Despite the limited number of donors in this study, we found the consistent stimulation of SCFA and the directive antimicrobial effect towards potentially harmful microbes to be remarkable. Prebiotic stimulation of western gut microbiotas is generally complicated by a large interindividual variability [52] and seems to be dependent on the initial microbial composition and richness [53,54]. We propose that the directional effects were the result of a synergistic effect of the different nutrients in the FCP. Cranberry polyphenols are widely recognized for their strong antimicrobial potency towards pathogens [39,40]. Recent studies also suggested cranberry oligosaccharides may exert antimicrobial and anti-adhesive effects against pathogens [55,56]. Indeed, comparative screening of cranberry polyphenol-rich fractions and cranberry fibrous fractions on the microbiota of obese mice demonstrated that both fractions act synergistically, decreasing levels of obesity-related pathobionts [51]. Cranberry fibers may further direct the gut microbial community by stimulating polyphenols-degrading families [51]. Finally, other cranberry nutrients may have added to the general reduction in potentially harmful microbes. We previously identified cranberry-derived salicylate and ß-resorcylate as contributors to reductions of *Enterobacteriaceae* levels in dysbiotic gut microbiota in vitro [16]. Hence, we propose that application of a whole cranberry (food) product containing different, synergistic gut microbiota modulating nutrients might hold a higher and more consistent therapeutic potential than its separate fractions.

Our data support the hypothesis that potential cranberry effects on systemic inflammation and infection may be modulated by gut microbiota, and more specifically microbial parameters that are associated with gut integrity, such as SCFA and mucin-associated or adhesive/invasive microbes. In our study, FCP decreased the abundance of a variety of phylogenetically diverse opportunistic pathogens, belonging to *Proteobacteria* as well as *Synergistetes*, but more species could be targeted. For example, in a placebo-controlled clinical trial with women suffering from recurrent urinary tract infection, Straub et al. [15] found that daily cranberry juice consumption specifically decreased just one *Flavonifractor* species. This species harbored the capacity to metabolize and transport tryptophan, a product that is known to modulate a variety of host functions beyond the gut, including nervous and immune systems [57]. Another target we could not confirm in this study, is cranberry stimulation of the mucin-degrading species *Akkermansia*. In mice models, cranberry products were found to protect from diet-induced obesity and other markers of the metabolic syndrome, and this was associated with higher levels of the *Akkermansia* species [13,51]. In our study, higher abundances of *Verrucomicrobiaceae* upon FCP dosing were only found for 1 donor. We concur with Anhê et al. (2015) [13] that cranberry fractions may influence *Akkermansia* levels through a combination of direct effects on gut microbiota and indirect effects on the host, for example by stimulating the epithelial production of its main carbon and energy source, mucin [58]. We therefore recommend that future in vitro studies investigating the effect of FCP and cranberry fractions take into account interactions with the gut mucosal interface.

A few limitations of this study should be addressed. First, use of highly complex models such as the M-SHIME simulating the colon microbiota via different regions (proximal and distal colon), each consisting a luminal and mucosal microbiota [59] limited the number of donors that can be tested while inclusion of parallel control arms is not economically feasible. This absence of a parallel control arms can be justified by the fact that the M-SHIME model involves a high number of quality control actions during its operation, thus ensuring stability over time. A second remark should be made concerning the absence of upper digestive and absorptive processes. Hence, the findings should be interpreted as effects that the FCP could have upon being dosed in a colon targeted formulation (e.g., capsule). Prior to moving into clinical trials, FCP upper digestion and absorption should be addressed in order to determine its most effective form and dosage of application. Finally, FCP effects on the microbiota of the M-SHIME were determined as shifts in relative abundances as no absolute quantification of the microbiota was performed. This is a limitation when testing test products that affect total bacterial load (e.g., antibiotics or prebiotics that respectively decrease and increase bacterial load). When testing the impact of providing additional nutrients (as was the case during the current project), bacterial load typically increases so that proportional data mask the impact on groups that are only mildly stimulated. In this specific project, it is possible that the strong increase of Bacteroidetes upon FCP supplementation could have masked a less pronounced effect on of FCP on *Bifidobacteriaceae*. While stimulated by FCP during the batch incubations (as detected with qPCR which is a quantitative method), *Bifidobacteriaceae* levels were not consistently impacted during the M-SHIME experiment. This highlights the importance of quantitative microbiome profiling as is preferentially performed through flow cytometry as described by Vandeputte et al. (2017) [60].

## 5. Conclusions

In conclusion, we demonstrated that FCP repeatedly and consistently boosted production of health-related acetate, propionate and butyrate in two in vitro models of the colon microbiota. 16S rRNA gene profiling demonstrated the consistent stimulation of *Bacteroidaceae* and *Prevotellaceae*. Further, we confirmed the potential of FCP to inhibit potentially harmful microbes, such as members of the *Pseudomonodaceae*, *Synergistetes* and *Enterobacteriaceae* families, despite interpersonal varying effects on the commensal colon microbial community. Overall, freeze-dried cranberry powder (FCP) exerted prebiotic potential in accordance with the latest consensus definition [61] given the selective utilization of FCP by host microorganisms and potential health-related effects. Future research should explore the underlying mechanisms with fecal samples from more donors, and particularly focus on the host-microbe colonic interface.

## Figures and Tables

**Table 1:** Composition of the freeze-dried whole cranberry powder (FCP). PAC: proanthocyanidin; BL-DMAC: Brunswick Labs 4-dimethylaminocinnamaldehyde method, with procyanidin A2 dimer as standard; OSC DMAC: Ocean Spray Cranberries 4-dimethylaminocinnamaldehyde method, with proprietary PAC extract from cranberries as standard.

Components	Freeze-Dried Whole Cranberry Powder (FCP)
total anthocyanins (mg/g)	5.98
organic acids (%)	22.68
flavonols (mg/g)	9.01
phenolic acids (mg/g)	1.81
PAC—BL DMAC (mg/g)	8.77
PAC—OSC DMAC (mg/g)	31.20
total phenolics (mg/g)—measured by Folin-Ciocalteu method	28.35

**Table 2:** Effect of FCP on microbial composition (families belonging to specific phyla) in the lumen (as estimated via 16S rRNA gene profiling (%)) of the simulated proximal and distal colon of the triple-M-SHIME. The data is presented as the difference between the value obtained at the end of the treatment period (TR: Day 35) and the end of the control period (C: Day 14) (*n* = 1). An increase (indicated in bold when > 2%) thus reflects a stimulation of a given family by repeated FCP treatment, while a decrease (indicated by underlining when < −2%) suggests a decrease due to FCP treatment.

Phylum	Family	Proximal Colon			Distal Colon		
		Donor 2	Donor 3	Donor 4	Donor 2	Donor 3	Donor 4
*Actinobacteria*	*Bifidobacteriaceae*	−6.4%	0.5%	−1.3%	1.2%	−1.1%	−0.1%
	*Coriobacteriaceae*	0.0%	0.1%	0.0%	0.3%	0.1%	0.7%
*Bacteroidetes*	*Bacteroidaceae*	**7.2%**	0.6%	0.0%	−8.3%	−17.6%	−29.4%
	*Bacteroidales_S24-7_group*	0.0%	0.0%	0.0%	0.0%	0.5%	0.3%
	*Porphyromonadaceae*	0.0%	0.0%	0.0%	-1.8%	−0.5%	−2.3%
	*Prevotellaceae*	0.0%	**24.7%**	**20.1%**	0.0%	**19.0%**	**12.6%**
	*Rikenellaceae*	0.0%	0.0%	0.0%	1.7%	0.0%	0.0%
*Firmicutes*	*Acidaminococcaceae*	**19.6%**	**2.5%**	**7.4%**	**2.8%**	**3.6%**	**7.2%**
	*Christensenellaceae*	0.0%	0.0%	0.0%	0.1%	0.0%	0.0%
	*Clostridiaceae_1*	0.0%	−0.7%	−1.3%	0.0%	0.0%	0.0%
	*Erysipelotrichaceae*	0.0%	0.0%	0.0%	0.1%	0.0%	0.2%
	*Eubacteriaceae*	0.0%	0.0%	0.0%	0.5%	0.1%	0.3%
	*Lachnospiraceae*	−3.5%	1.6%	**2.1%**	**11.9%**	0.1%	**2.2%**
	*Ruminococcaceae*	0.0%	0.0%	0.0%	**2.8%**	1.5%	**4.2%**
	*Veillonellaceae*	−8.3%	−18.6%	−15.5%	−5.7%	−2.7%	−3.2%
*Lentisphaerae*	*Victivallaceae*	0.0%	0.0%	0.0%	-0.2%	−0.2%	−0.2%
*Proteobacteria*	*Alcaligenaceae*	0.0%	0.6%	0.2%	0.0%	0.2%	0.2%
	*Campylobacteraceae*	0.0%	0.0%	0.0%	0.0%	0.0%	0.0%
	*Desulfovibrionaceae*	0.0%	0.1%	0.0%	0.4%	−0.5%	−2.0%
	*Enterobacteriaceae*	−4.9%	−2.5%	−3.3%	0.0%	−0.1%	-0.1%
	*Pseudomonadaceae*	−1.8%	−8.1%	−4.1%	−2.7%	−2.9%	−4.3%
	*Rhodospirillaceae*	0.0%	0.0%	0.0%	0.2%	0.3%	**2.4%**
	*Xanthomonadaceae*	−1.9%	−0.8%	−4.2%	−0.1%	−0.1%	−0.3%
*Synergistetes*	*Synergistaceae*	0.0%	0.0%	0.0%	−4.2%	**4.6%**	−3.4%
*Verrucomicrobia*	*Verrucomicrobiaceae*	0.0%	0.0%	0.0%	0.7%	−4.2%	**15.2%**

## Supplementary Materials

The following are available online at https://www.mdpi.com/article/10.3390/biotech11020014/s1. Figure S1: Effect of FCP on levels of acetate (A), propionate (B), butyrate (C), bCFA (D), total SCFA (E) and *Bifidobacteriaceae* (F), shown for each individual donor, Figure S2: Effect of fermentation of FCP on SCFA levels in the simulated proximal and distal colon for donors 2 and 4. Table S1: Effect of FCP on microbial composition (families belonging to specific phyla) in the simulated mucus, Table S2: Microbial composition (families belonging to specific phyla) in the lumen of the triple-M-SHIME during the control period, Table S3: Microbial composition (families belonging to specific phyla) in the simulated mucus of the triple-M-SHIME during the control period.
