# Supplementary material for: A Freeze-Dried Cranberry Powder Consistently Enhances SCFA Production and Lowers Abundance of Opportunistic Pathogens In Vitro
Source: BioTech (Basel). 2022 May 6;11(2):14. doi: 10.3390/biotech11020014 (PMC9264401; doi:10.3390/biotech11020014)
Supplement: Supplementary file 1 [file biotech-11-00014-s001.zip › biotech-1654117-supplementary.pdf]

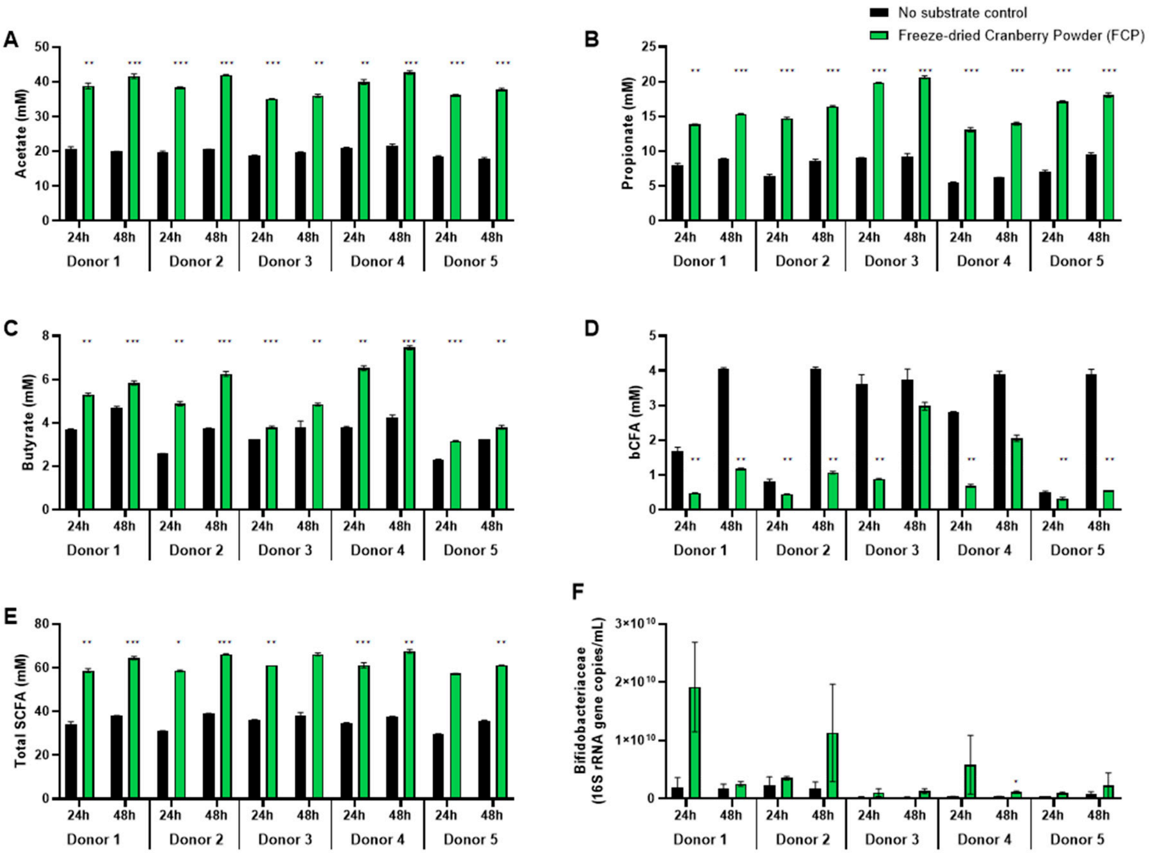

Figure S1. Effect of FCP on average (± SD) levels of acetate (A), propionate (B), butyrate (C), bCFA (D), total SCFA (E) and *Bifidobacteriaceae* (F), upon incubation with human faecal microbiota derived from 5 different human donors. All conditions were tested in technical duplicate. The data is presented as the average (± SD) for each of the different test conditions (n = 2). Statistical differences due to treatment were indicated by */**/*** (0.01 < adjusted p-value < 0.05/0.001 < adjusted p-value < 0.01/ adjusted p-value < 0.001).

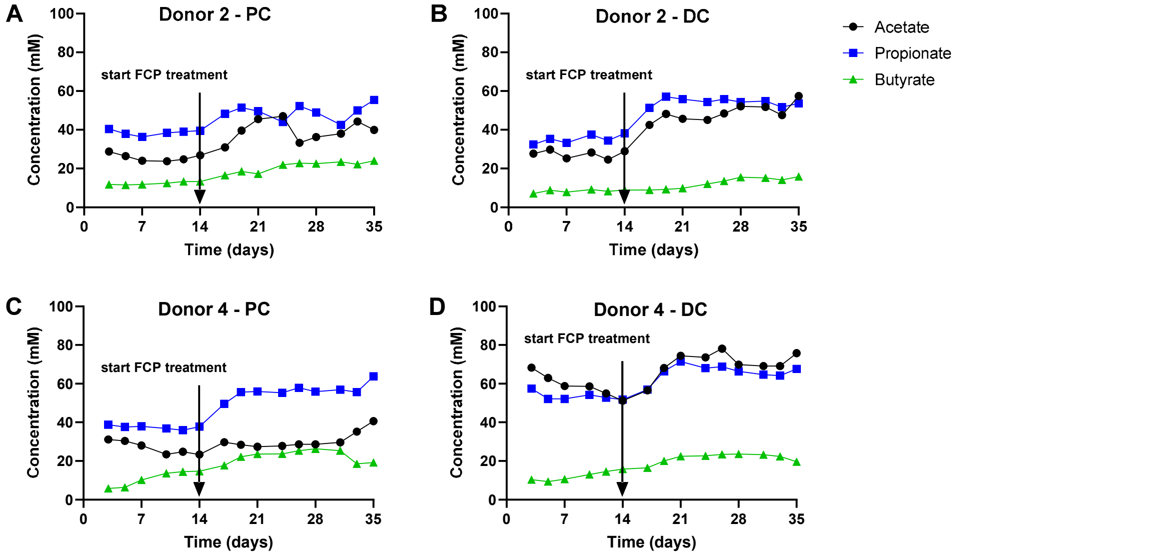

Figure S2. Effect of fermentation of FCP on SCFA levels (mM) in the simulated proximal colon (A/C) and distal colon (B/D) during both the control (Days 0–14) and treatment period (Days 14–35) of the triple-M-SHIME for donors 2 (A/B) and 4 (C/D). Arrows indicate the start of the FCP treatment.

Table S1. Effect of FCP on microbial composition (families belonging to specific phyla) in the simulated mucus (as estimated via 16S rRNA gene profiling (%)) of the simulated proximal and distal colon of the triple-M-SHIME. The data is presented as the difference between the value obtained at the end of the treatment period (TR: Day 35) and the end of the control period (C: Day 14) (n = 1). An increase (indicated in bold when > 2 %) thus reflects a stimulation of a given family by repeated FCP treatment, while a decrease (indicated by underlining when < -2 %) suggests a decrease due to FCP treatment.

Phylum	Family	Proximal colon			Distal colon		
		Donor 2	Donor 3	Donor 4	Donor 2	Donor 3	Donor 4
Actinobacteria	*Bifidobacteriaceae*	-13.1%	0.0%	-34.8%	**6.1%**	0.4%	**4.6%**
	Coriobacteriaceae	0.1%	0.8%	0.0%	**2.6%**	0.2%	**2.5%**
Bacteroidetes	*Bacteroidaceae*	**22.4%**	**6.1%**	0.6%	1.5%	-7.4%	-7.8%
	Bacteroidales_S24-7_group	0.0%	0.0%	0.0%	0.0%	**4.9%**	2.0%
	Porphyromonadaceae	0.0%	0.4%	0.0%	1.3%	-2.2%	0.1%
	Prevotellaceae	0.0%	**5.3%**	**16.7%**	0.0%	**2.4%**	**3.3%**
	Rikenellaceae	0.0%	0.1%	0.0%	-0.5%	0.1%	0.4%
Firmicutes	*Acidaminococcaceae*	**9.4%**	-1.7%	**10.5%**	**4.2%**	**3.6%**	**2.3%**
	Christensenellaceae	0.0%	0.0%	0.0%	0.0%	0.0%	0.1%
	Clostridiaceae_1	-0.4%	-0.3%	-0.4%	-0.4%	0.6%	-0.7%
	Erysipelotrichaceae	0.0%	0.0%	0.0%	0.4%	0.4%	**3.6%**
	Eubacteriaceae	0.0%	0.0%	0.0%	-0.8%	**2.1%**	0.3%
	Lachnospiraceae	-20.2%	**3.4%**	1.5%	**7.5%**	**7.6%**	-3.3%
	Ruminococcaceae	0.0%	0.0%	0.0%	0.2%	0.9%	1.5%
	Veillonellaceae	**6.8%**	-11.3%	**2.3%**	-0.5%	-0.5%	1.2%
Lentisphaerae	*Victivallaceae*	0.0%	0.0%	0.0%	-0.1%	0.3%	0.8%
Proteobacteria	*Alcaligenaceae*	0.0%	-0.2%	0.5%	0.0%	0.3%	0.3%
	Campylobacteraceae	-0.4%	0.0%	0.0%	0.0%	0.0%	0.0%
	Desulfovibrionaceae	0.0%	0.7%	0.0%	-1.2%	-0.9%	-1.8%
	Enterobacteriaceae	-3.9%	-1.6%	**4.2%**	0.1%	0.5%	-0.2%
	Pseudomonadaceae	-0.7%	-2.2%	-0.5%	0.8%	-0.1%	-0.1%
	Rhodospirillaceae	0.0%	0.1%	0.0%	0.3%	0.2%	1.5%
	Xanthomonadaceae	-0.4%	-0.1%	-0.6%	0.0%	0.0%	-0.1%
Synergistetes	*Synergistaceae*	0.0%	0.3%	0.0%	-21.0%	-12.8%	-11.4%
Verrucomicrobia	*Verrucomicrobiaceae*	0.0%	0.0%	0.0%	-0.1%	-0.4%	0.9%

Table S2. Microbial composition (families belonging to specific phyla) in the lumen (as estimated via 16S rRNA gene profiling (%)) of the simulated proximal and distal colon of the triple-M-SHIME during the control period (Day 14) (n = 1).

Phylum	Family	Proximal colon			Distal colon		
		Donor 2	Donor 3	Donor 4	Donor 2	Donor 3	Donor 4
Actinobacteria	*Bifidobacteriaceae*	22.5%	2.0%	1.8%	0.4%	1.8%	0.6%
	Coriobacteriaceae	0.0%	0.0%	0.0%	0.2%	0.3%	0.1%
Bacteroidetes	*Bacteroidaceae*	19.2%	1.0%	0.0%	45.3%	39.6%	43.8%
	Bacteroidales_S24-7_group	0.0%	0.0%	0.0%	0.0%	0.2%	0.2%
	Porphyromonadaceae	0.0%	0.0%	0.0%	2.5%	1.4%	3.6%
	Prevotellaceae	0.0%	34.6%	27.8%	0.0%	8.1%	5.2%
	Rikenellaceae	0.0%	0.0%	0.0%	0.2%	0.2%	0.1%
Firmicutes	*Acidaminococcaceae*	1.8%	4.1%	5.4%	1.3%	1.8%	1.6%
	Christensenellaceae	0.0%	0.0%	0.0%	0.0%	0.0%	0.0%
	Clostridiaceae_1	0.1%	0.8%	1.7%	0.0%	0.0%	0.0%
	Erysipelotrichaceae	0.0%	0.0%	0.0%	0.0%	0.0%	0.0%
	Eubacteriaceae	0.0%	0.0%	0.0%	0.1%	0.3%	0.2%

	Lachnospiraceae	8.6%	1.9%	0.2%	19.7%	18.3%	15.4%
	Ruminococcaceae	0.0%	0.0%	0.0%	0.5%	0.5%	0.3%
	Veillonellaceae	37.1%	43.6%	49.6%	10.3%	10.1%	12.2%
Lentisphaerae	*Victivallaceae*	0.0%	0.0%	0.0%	0.4%	0.3%	0.3%
Proteobacteria	*Alcaligenaceae*	0.0%	0.4%	0.0%	0.3%	0.2%	0.1%
	Campylobacteraceae	0.1%	0.0%	0.0%	0.0%	0.0%	0.0%
	Desulfovibrionaceae	0.0%	0.0%	0.0%	0.7%	1.5%	2.4%
	Enterobacteriaceae	6.5%	2.6%	4.7%	0.1%	0.1%	0.2%
	Pseudomonadaceae	1.9%	8.2%	4.7%	4.2%	3.2%	5.2%
	Rhodospirillaceae	0.0%	0.0%	0.0%	0.1%	0.2%	0.4%
	Xanthomonadaceae	2.0%	0.8%	4.3%	0.1%	0.1%	0.3%
Synergistetes	*Synergistaceae*	0.0%	0.0%	0.0%	11.2%	7.0%	7.4%
Verrucomicrobia	*Verrucomicrobiaceae*	0.0%	0.0%	0.0%	2.1%	4.7%	0.1%

Table S2. Microbial composition (families belonging to specific phyla) in the simulated mucus (as estimated via 16S rRNA gene profiling (%)) of the simulated proximal and distal colon of the triple-M-SHIME during the control period (Day 14) (n = 1).

**Phylum**	**Family**	**Proximal colon**			**Distal colon**		
		**Donor 2**	**Donor 3**	**Donor 4**	**Donor 2**	**Donor 3**	**Donor 4**
Actinobacteria	*Bifidobacteriaceae*	43.2%	42.0%	60.4%	2.4%	6.9%	2.0%
	Coriobacteriaceae	0.0%	0.3%	0.0%	0.6%	1.3%	0.6%
Bacteroidetes	*Bacteroidaceae*	6.3%	0.5%	0.0%	12.5%	21.2%	16.9%
	Bacteroidales_S24-7_group	0.0%	0.0%	0.0%	0.0%	1.0%	1.1%
	Porphyromonadaceae	0.0%	0.0%	0.0%	1.5%	3.2%	2.0%
	Prevotellaceae	0.0%	7.2%	3.1%	0.0%	0.4%	0.3%
	Rikenellaceae	0.0%	0.0%	0.0%	1.7%	0.5%	1.6%
Firmicutes	*Acidaminococcaceae*	1.6%	16.7%	6.8%	0.9%	2.4%	2.1%
	Christensenellaceae	0.0%	0.0%	0.0%	0.3%	0.1%	0.0%
	Clostridiaceae_1	0.9%	0.5%	1.5%	0.9%	0.1%	1.2%
	Erysipelotrichaceae	0.0%	0.0%	0.0%	0.3%	0.1%	0.2%
	Eubacteriaceae	0.0%	0.0%	0.0%	2.5%	0.4%	1.0%
	Lachnospiraceae	26.3%	4.9%	0.8%	33.3%	22.6%	37.2%
	Ruminococcaceae	0.0%	0.0%	0.0%	3.2%	1.9%	3.2%
	Veillonellaceae	15.8%	21.5%	23.9%	4.2%	5.4%	4.1%
Lentisphaerae	*Victivallaceae*	0.0%	0.0%	0.0%	0.6%	0.2%	0.3%
Proteobacteria	*Alcaligenaceae*	0.0%	1.7%	0.2%	0.4%	0.4%	0.2%
	Campylobacteraceae	0.5%	0.0%	0.0%	0.0%	0.0%	0.0%
	Desulfovibrionaceae	0.0%	0.0%	0.0%	2.1%	2.0%	2.5%
	Enterobacteriaceae	4.2%	2.3%	1.7%	0.1%	0.1%	0.5%
	Pseudomonadaceae	0.7%	2.2%	0.8%	0.0%	0.2%	0.1%
	Rhodospirillaceae	0.0%	0.0%	0.0%	0.1%	0.2%	0.4%
	Xanthomonadaceae	0.4%	0.1%	0.8%	0.0%	0.0%	0.1%
Synergistetes	*Synergistaceae*	0.0%	0.2%	0.0%	31.1%	28.3%	21.8%
Verrucomicrobia	*Verrucomicrobiaceae*	0.0%	0.0%	0.0%	0.2%	0.5%	0.0%
